# Flow cytometric data analysis of circulating progenitor cell stability

**DOI:** 10.1016/j.dib.2016.11.050

**Published:** 2016-12-07

**Authors:** Ernestine A. Mahar, Liping Mou, Salim S. Hayek, Arshed A. Quyyumi, Edmund K. Waller

**Affiliations:** Emory University School of Medicine, Atlanta, GA, USA

## Abstract

A recent publication by Mekonnen et al. demonstrated that among women with non-obstructive coronary artery disease, higher levels of circulating progenitor cells in the blood (CPC), were associated with impaired coronary flow reserve [1].

We performed a quality control assessment of the stability of circulating blood progenitor cells in blood samples stored at 4 °C, to determine the time period during which blood samples can be analyzed and yield consistent data for progenitor cell content. Healthy volunteers (*n*=6) were recruited and underwent phlebotomy, and blood was stored in EDTA tubes at 4 °C. Flow cytometry was performed to quantitate progenitor cell subsets at 0–4 h, 24 h, and 48 h post phlebotomy. All processed samples were fixed with 1% Paraformaldehyde and 1,000,000 total data events were collected. We found no significant differences in PC data for both CD34+ (*P*=0.68 for one-way ANOVA) and CD34+/CD133+ (*P*=0.74 for one-way ANOVA).

**Specifications Table**

**Table:** 

Subject area	*Medicine*
More specific subject area	*Cardiology*
Type of data	*Tables, graphs*
How data was acquired	*Flow cytometry on BD FACS Canto II RUO*
Data format	*Analyzed*
Experimental factors	*EDTA preserved samples*
Experimental features	*A lyse-no wash procedure with the addition of fluorescent counting beads*
Data source location	*Atlanta, GA, USA.*
Data accessibility	*Data included in the article*

**Value of the data**

• Increased confidence in data from rare progenitors in blood samples stored up to 48 h.
• Increased opportunities for collaborations with distant institutions up to 48 h shipping samples to a central lab for analysis.
• Multiple samples collected during one day can be analyzed in a batch the following day, thus increasing the efficiency of laboratory personnel analyzing samples.

## 1 Data

**Fig. 1:**
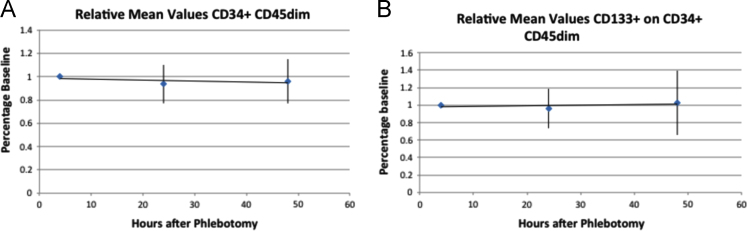
Stability of progenitor cells over time during storage at 4 °C. A. Percentage change in mean values of CD34+/CD45^dim^ cells from baseline values measured at 0–4 h and after 24 and 48 h storage. B. Percentage change of mean values of CD34+/CD45^dim^/CD133+ from baseline values measured at 0–4 h and after 24 and 48 h storage.

Progenitor cell content for CD34+/CD45^dim^, CD34+/CD133+/CD45^dim^, subsets in 300 uL aliquots of anticoagulated blood were measured by flow cytometry. Triplicate aliquots of blood from each sample were analyzed at each time point, and the mean values for each time point for every subject were calculated to determine the stability of the progenitor cell content during storage (Fig. 1). The standardized mean values for the 0–4 h time point was used as the baseline, and the relative change of mean values for the subsequent time points was calculated as a percentage of the baseline value. We found no significant differences in PC counts for both CD34+ (Fig. 1 Panel A, *P*=0.68) and CD34+/CD133+ (Fig. 1 Panel B, *P*=0.74).

## 2 Experimental design, materials and methods

Gently mix by inversion and reverse pipet 300 ul blood sample to a 5 ml FACS tube. Add antibody cocktail to blood sample and vortex and incubate in the dark for 15 min. Add 1.2 ml Ammonium chloride lysis buffer, vortex and incubate in the dark for 10 min. Sample should become relatively transparent post lysis. Add 1.2 ml staining media the add 350 ul 1% paraformaldehyde to fix cells, seal the tubes with parafilm and mix gently by inverting several times. Store samples at 4 °C. Before acquisition on the FACS Canto II, reverse pipet 100 ul Invitrogen counting beads to the prepared samples, mixed gently and run. FCS files we a’re analyzed in FlowJo version 9.8.5.

## 3 Materials

**Table:** 

Item	Manufacturer	Catalog number
CD34	Becton Dickson	340430
CD133	Miltenyi Biotech	130-090-826
CD45	Becton Dickson	348805
AccuCheck Counting beads	Fisher	PCB100_3654889900
Tris Hydrochloride	Fisher	BP153
Ammonium chloride	Sigma	A4514
EDTA	Sigma	ED2SS
Ammonium hydroxide	Sigma	A6899
Phosphate buffered saline (PBS)	Corning	21–040-CV
Fetal Bovine Serum (FBS)	Sigma	F4135
Sodium Azide	Sigma	S8032
Paraformaldehyde	Fisher	04042

Tris Buffered Ammonium chloride Lysis solution-

2.06 g Tris Hydrochloride.

8.26 g Ammonium chloride.

0.037 g EDTA.

QS to 1 L DI H_2_O and pH to 7.2–7.5 using ammonium Hydroxide. Store at RT.

Staining Media-

1X PBS 485 mL.

15 mL FBS.

0.5 g of Sodium azide (NaN3).

Combine and store at 4 °C.
