# Supplementary material for: Flow cytometric data analysis of circulating progenitor cell stability
Source: Data Brief. 2016 Dec 7;10:346–8. doi: 10.1016/j.dib.2016.11.050 (PMC5157704; doi:10.1016/j.dib.2016.11.050)
Supplement: Supplementary file 1 — Supplementary material [file mmc1.docx]

10/12/16

To whom it may concern,

The authors on the data in brief article titled Flow Cytometric Data Analysis of Circulating Progenitor Cell Stability and under ID number DIB-D-16-00659 do not have any conflicts of interest that require disclosure.

Sincerely,

Ernestine Mahar
